# Monthly Summary

**Published:** 1899-02

**Authors:** 


### Monthly Summary.

A NEW COUNTER-DIE.—A few weeks ago I had some trouble when making a seamless crown in using lead as a female die, as the lead stuck to the crown badly. I wondered how modeling composition would work. I took some quick hard-setting composition and warmed it as usual. I then forced it in an old brass bell and forced the zinc die into it and cooled under water. I think it better than lead. I have used it several times with success.—J. T. Wheelock, in *Dental Brief.*

REPAIRING GOLD FILLINGS.—I have no means of knowing what is the general practice—whether it is that of removing the whole gold so as to get access to the defective place, or that simply cutting out the decay and repairing with gold or amalgam, or even with gutta-percha. My own practice has been almost invariably that of repairing the defective place, if the body of the filling is good. If the gum cannot be held away by the rubber-dam, it can be by a suitably shaped instrument, so that access can be had to the cavity from both the buccal and lingual sides, and the operation becomes a very simple one either for the use of gold or amalgam.

If a large gold filling requires repairing at this point, it is more satisfactory to do it with gold if it can be done accurately, but if the defective place is inaccessible, a thoroughly durable operation can be made with amalgam. I consider this one of the best uses to which amalgam can be put. I have so many with it in this way, and have seen them last so many years, that I have come to have great confidence in it for these places. The result is somewhat like that attained by using amalgam in the upper half or two-thirds of the cavity, and finishing with gold at the same sitting.

The repairs I have in mind are only slight. however, and do not let the amalgam come in sight. The ability to make these repairs so easily is one reason why I am not so ready now as formerly to cut the teeth away so that each cavity on the approximate surface shall go under the gum.—DR. STAFFORD, in *International*.

---

SETTING OF CROWNS.—Dr. Taggart fits gutta-percha onto the post and in the root, and gets a proper adaptation first; then he dries the roots out, and paints the inside of the canal with cajeput or eucalyptus; and he lets that dry a little, and paints the inside of that with chloro-percha; then he paints the gutta-percha on the post with chloro-percha, warms it a little, and drives it home. They have this advantage: They are not set solidly as they are with cement, and possibly the gutta-percha preventing a fracture of the porcelain.—*Dental Review*.

TIPPING TEETH.—A little practice that has been successful in my hands is in tipping teeth. I do hate to destroy live pulps, and where I find both corners are gone, and the tooth very frail, I grind the surface' beveling from the neck of the tooth down to the cutting-edge of the tooth on both sides I grind off a little to the cutting-edge, then use thin gold foil, and burnish that to the tooth on three surfaces, stiffen it with solder of 22-carat plate, solder it, and again grind and cement that in place. It looks very well, can hardly be detected from gold filling, and you have and edge that will protect the frail edges of enamel. It is only necessary to break away very little of the frail edge. You can burnish the gold over the frail edges with any amount of assurance, and as long as it stays there it will protect the edges of the enamel.—M. S. Smith, in *Cosmos*.

---

THE MEDICAL TREATMENT OF TOOTHACHE.—In a case of severe neuralgia of the lower maxilla, a local application of half a grain of cocain brought permanent relief, although it is not desirable to place the remedy in the patient's hand. The following prescription has proven itself very effective in those persons that have suffered for many sleeplcss nights :

| | | | |
|---|---|---|---|
| ℞ | Miniac sulf, | - - | grs. ij. |
| | Acid. hydrobrom, | - | gtt. v. |
| | Tinct. gelsem, | - | gtt. xv. |
| | Syr. simpl. | - - | ℥ ss. |
| | Ag. dist. ad: | - | ℥ j. |

The most effective remedy is salicylate of soda, even in those cases where pain results from "taking cold" and in the beginning of periostitis.

The dose should be 15 grains in combination with about 30 drops of tincture of belladonna, eventually repeated in about four hours.—Ash & Sons, "Vierteljahrs-Bericht.

REMOVAL OF BROKEN NERVE BROACHES AND DRILLS FROM ROOT CANALS.—Some months since, while using a Gates-Glidden drill fer enlarging the orifice of root-canal for treatment in a superior cuspid, the blades of the drill caught and cut a screw-thread in the tooth tissus; and the shank was twisted off instantly. I knew it was no use to drill by the side to remove it.

The cavity was treated with a small pledget of cotton wet with fifty per cent. sulphuric acid. sealed with gutta-percha for three or four days.

Next sitting, opened up cavity, found it filled with black oxide of steel, rinsed out, no discolor to dentin.

Found that a fine broach would pass by the drill. I gave another treatment. At next sitting the steel came out readily by the use of a Donaldson root cleaner.

Canal in fine order and not discolored.

Second trial is all I have had occasion to make. A barbed nerve broach gave out in the canal. No great effort was made at the time to remove it. Gave some treatment.

Next sitting pyrozon, three per cent., was injected to cleanse the canal, and to my great delight the broach appeared with the foam. In either case the steel showed but slight change, except in color.

This may be new to some others as to myself. I do not recollect of its being reported. All I claim for it is that it has served me well in two cases.—S. B. Palmer, in *Dental Weekly.*

---

AN EXACT METHOD FOR MEASURING RUBBER.—Take a common glass tumbler and a piece of wood long enough to reach across the top, In one side or edge of the wood drive a small wire nail, allowing the head to protrude about one-half inch.

Remove the wax from the investment and roll it in a ball. Insert the point of an instrument in the wax and place it in the tumbler. Lay the piece of wood across the tumbler, the nail down. Pour water in the glass until the surface touches the head of the nail. Remove the wax from the water and replace it with rubber until the surface of the water touches the nail, and the exact quantity of rubber required will be in the tumbler.

I have found this method to be quick and accurate. There is never any question about having the right quantity of rubber.—Dr, W. H. Craig, Oakland, California, in *Pacific Medico-Dental Gazette.*

---

A DENTIST FOR THE ARMY.—We are very glad to announce recently that a dentist had been requisitioned to attend to the teeth of some of the regiments in Egypt. It is the first step which costs, and this step in the right direction we hope will soon be followed by others. Apart from the extraction of teeth almost nothing is done for the care of the teeth by the army surgeon. While our army medical corps embraces men who are competent in the practice of medicine and surgery, yet the practice of dentistry is so highly specialized, requiring not only a mechanical bent of mind, but also a special training in operative work, that there is probably not one of that corps to-day professing a knowledge of dentistry. We are a practical people, and we think that when it is borne in upon the official mind that dentists are badly needed for the services, in course of time they will be supplied.—"Brit, Jour. Den. Science.

---

RELIEVING CARBOLIC ACID BURNS.—Carbolic acid burns resulting from the use of phenic or carbolic acid may be readily antagonized by the application of ethylic alcohol to the injured surface. Carbolic acid is freely dissolved in alcohol, and thereby its intensity is greatly diminished.—Dr. H. J. Wardlaw, in the "Weekly Dentist."

MATRICES.—At a recent meeting of the Chicago Dental Society the question of using matrices was discussed to a slight extent by some of the members. Surprise was expressed by one gentleman that an old practitioner had adopted the matrix at this late day. Matrices have been used in filling teeth for many years, especially when using plastics. In regard to the use of the matrix in gold filling there is still a division of opinion. Some contend that a matrix may prevent the perfect joint on the long axis of a tooth, but we believe that a little careful practice will soon make the indifferent operator a more careful and painstaking worker. The matrix reduces to a minimum the subsequent polishing of the filling, and if a matrix is carefully and firmly adjusted the contour is more easily made than without its use.—"Review."

---

LANCING ABSCESS.—When a young patient calls with the first molar dead in the inferior jaw, the face badly swollen and an abscess about to make an external opening, which would leave a scar that would mortify both patient and yourself, don't extract the tooth, in your eagerness to prevent the fistula. Insert a small bistoury along the external surface of the bone opposite the affected tooth and in the direction of the anticipated opening, not fearing to cut too deep. The abscess will open in the incision made and pain will soon subside and the tooth may be saved, were it possible to save the tooth before the abscess formed.—W. H. Weaver, in "Dental Weekly."

---

CONTOURING.—Tooth form should be held secondary to the contouring of the interproximal space, the guarding of which should be the first consideration. The form of the tooth or even its full occlusion, is of less importance. The health of the tooth and its surroundings is the first consideration.—E. V. Black, *Dental Review.*

PYORRHEA.—Dr. Pich before the Illinois Society said: Sulphate of copper is a useful agent in the treatment of pyorrhea, and it is also a favorite of mine in the treatment of abnormal swellings of the gums from whatever cause. The gums are dried, and copper applied by means of a piece of orange wood, whittled thin, which is first dipped in water, and passed into the copper a quantity of the powder will cling to the stick; then pack the copper down between the teeth and swollen gums. You can use it freely. It is not necessary to exercise care as to the quantity of the powder to be used; let it remain there for two or three minutes, then with a syringe of warm water wash the excess away. You will be surprised in the course of two or three days, and also much gratified, to see the extent to which the swollen gums have been reduced.—"Dental Weekly."

---

VAIN VERACITY.—Fired with zeal to emulate a great and good man, Alfred cut down the cherry tree with his little hatchet.

Then he went into the house and informed his stern parent that he could not tell a lie.

"Do you think I shall be the father of my country?" Alfred now asked anxiously.

"There is no certainty ahout it, my son," replied the old man, with streaming eyes. "Times have changed in 150 years. The boy who cannot tell a lie is assured of nothing except that he can't very well be a painless dentist."

Thus we see that opportunity is a large element in success.—*Detroit Free Press.*

